# Supplementary material for: Genome-wide association analysis implicates dysregulation of immunity genes in chronic lymphocytic leukaemia
Source: Nat Commun. 2017 Feb 6;8:14175. doi: 10.1038/ncomms14175 (PMC5303820; doi:10.1038/ncomms14175)
Supplement: Supplementary Information — Supplementary Figures, Supplementary Tables, Supplementary References. [file ncomms14175-s1.pdf]

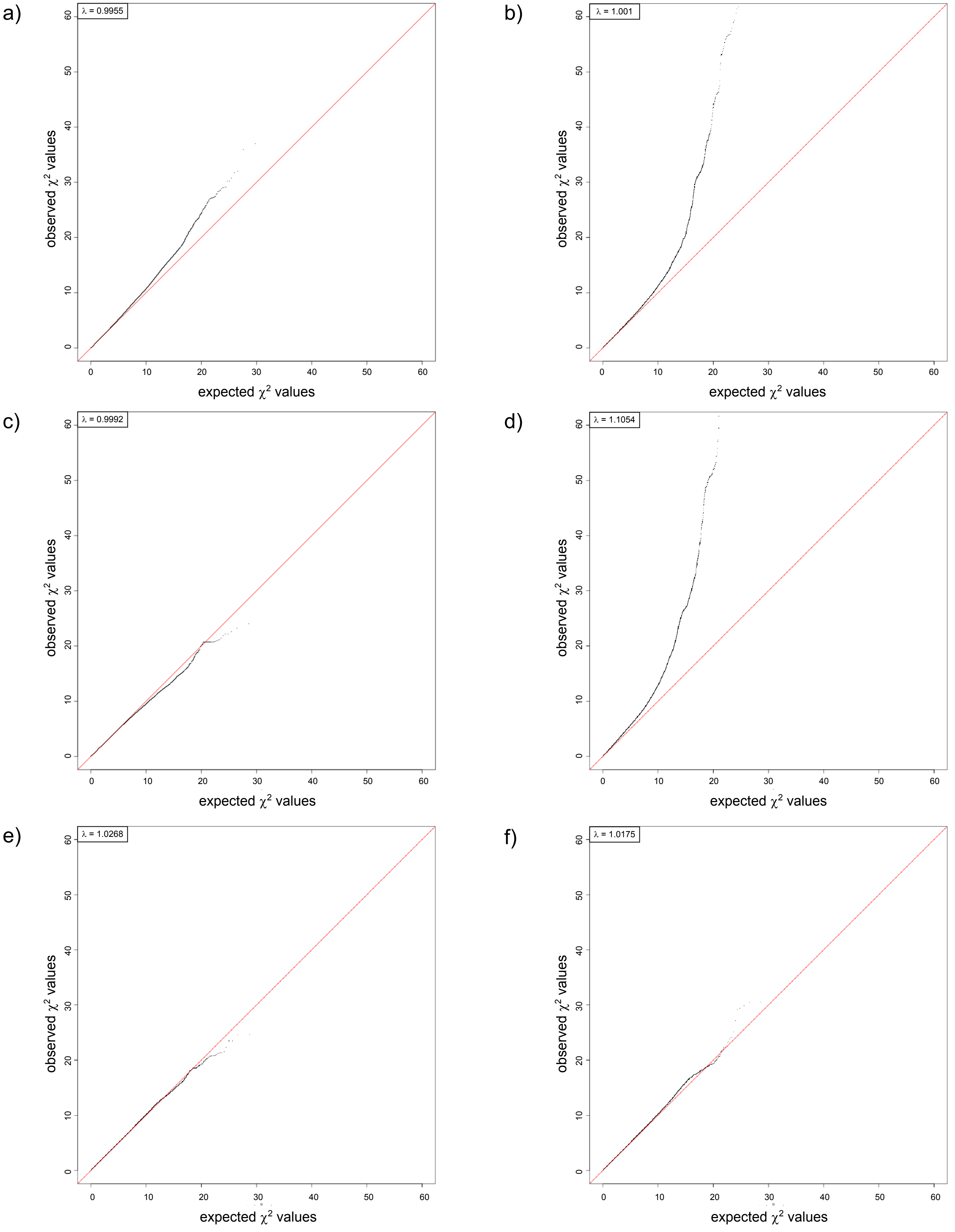

Supplementary Figure 1: Quantile-Quantile (Q-Q) plots of observed and expected χ^2 values of association between SNP genotype and risk of chronic lymphocytic leukemia.
a) UK-CLL1, b) UK-CLL2, c) GEC, d) NHL GWAS, e) UCSF and f) Utah. The red line represents the null hypothesis of no true association.

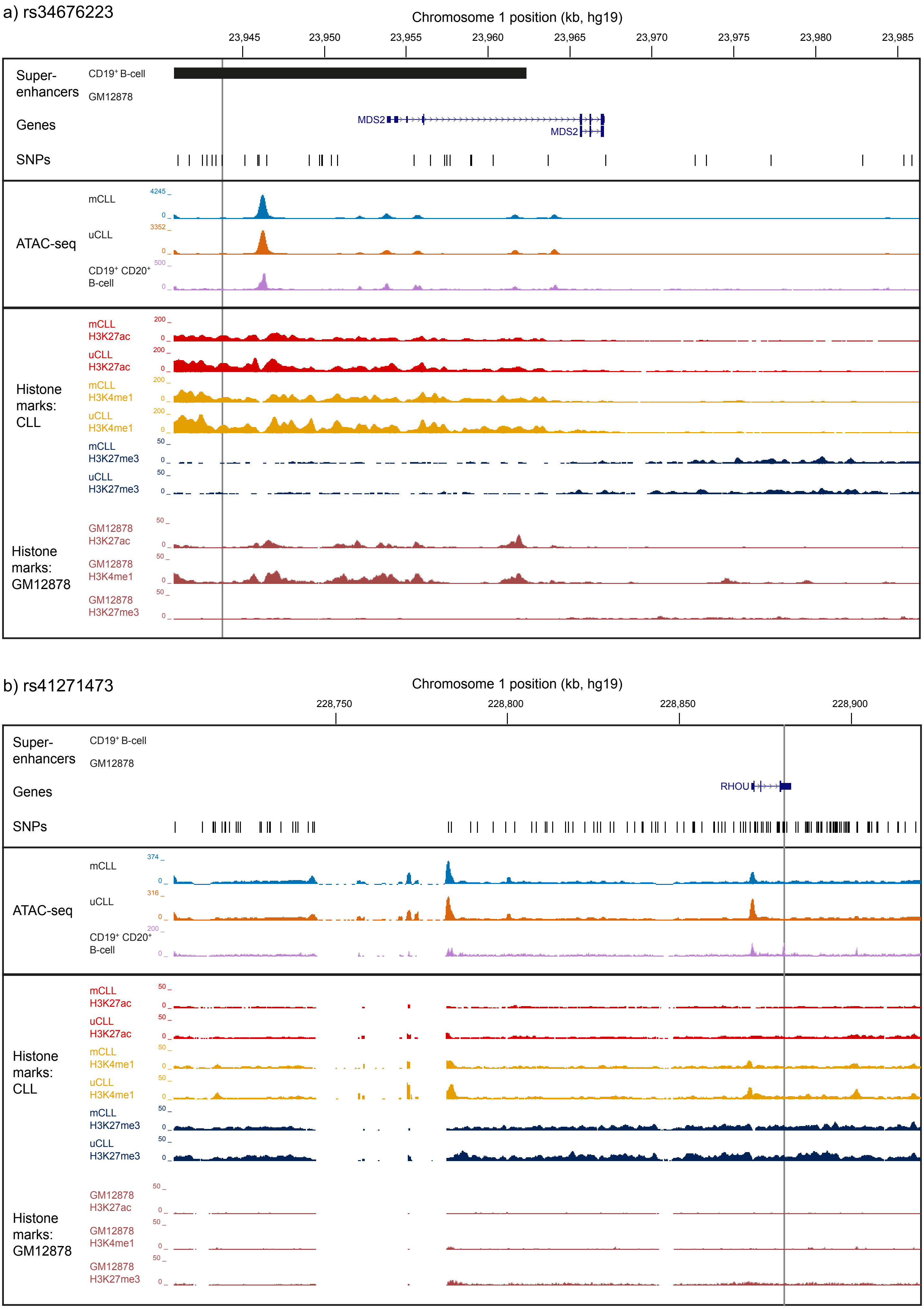

Supplementary Figure 2

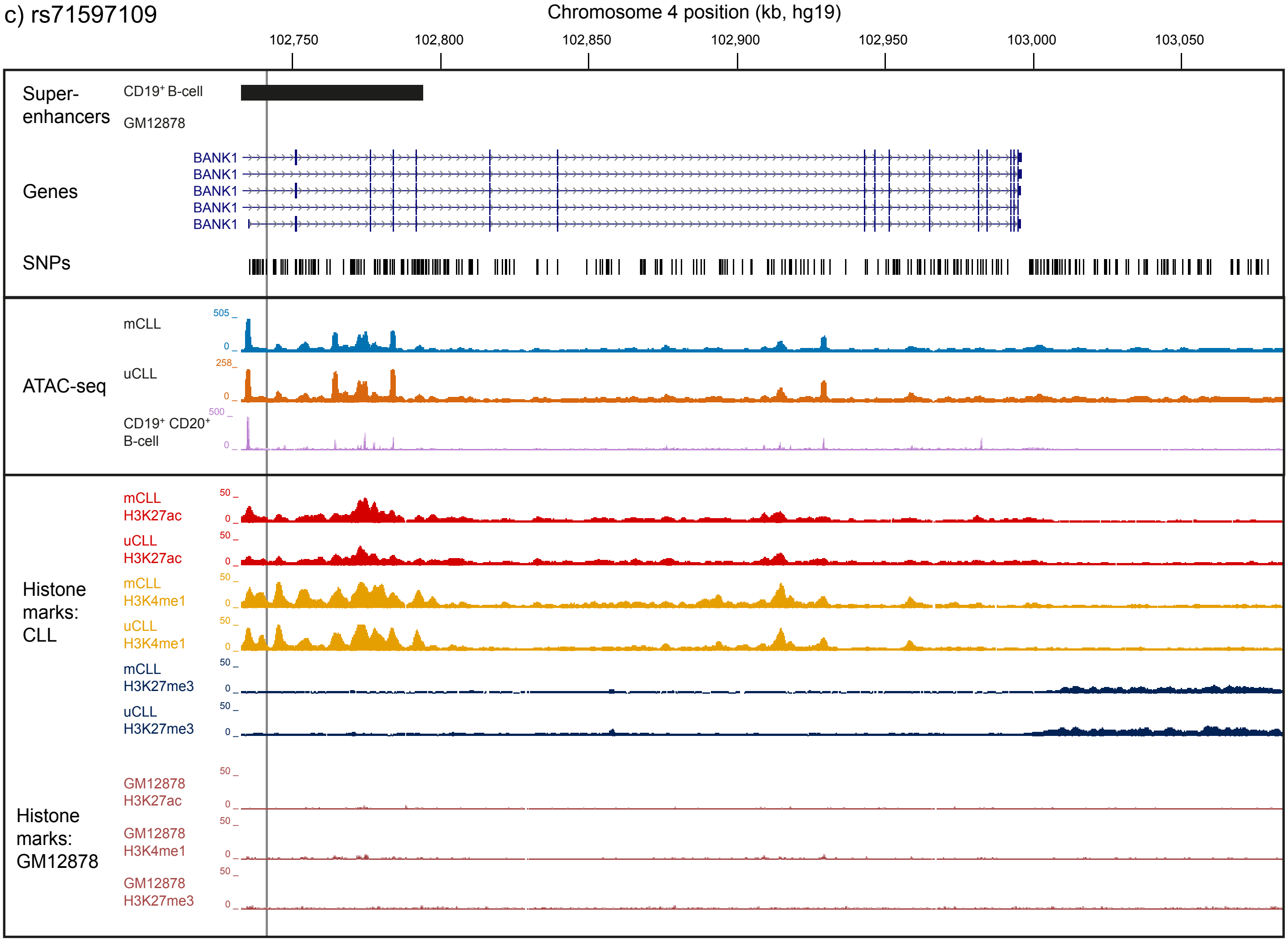

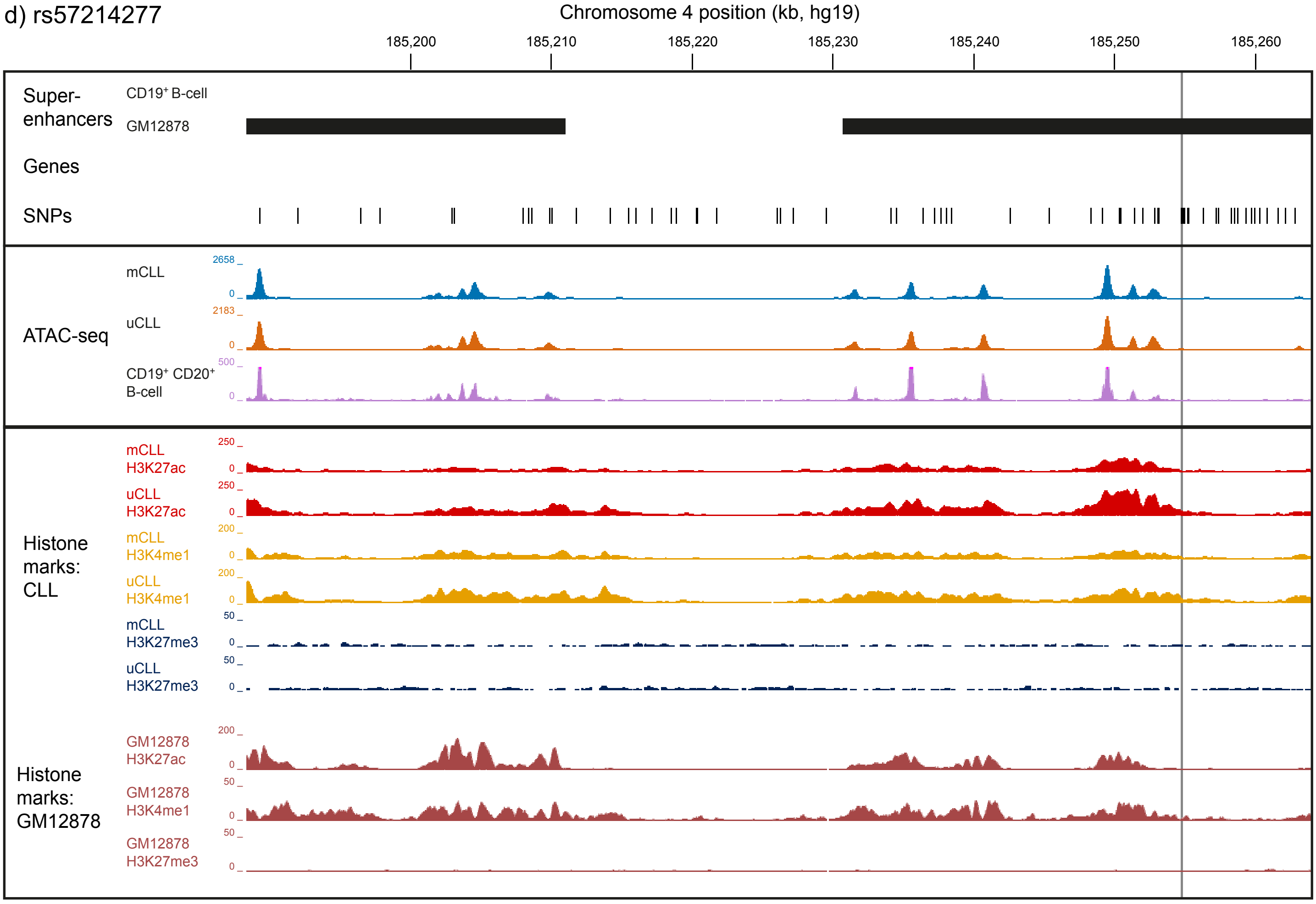

Supplementary Figure 2

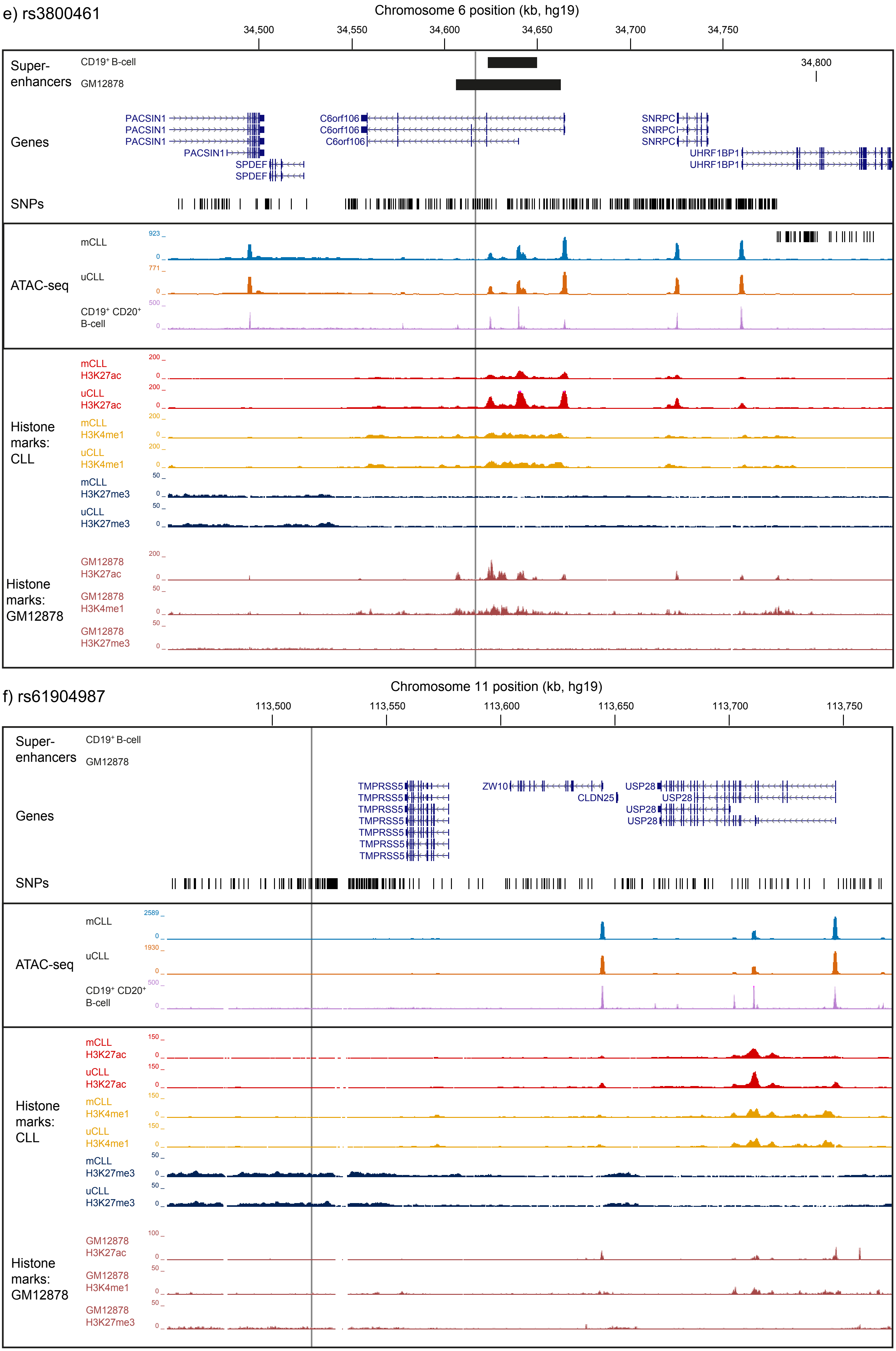

Supplementary Figure 2

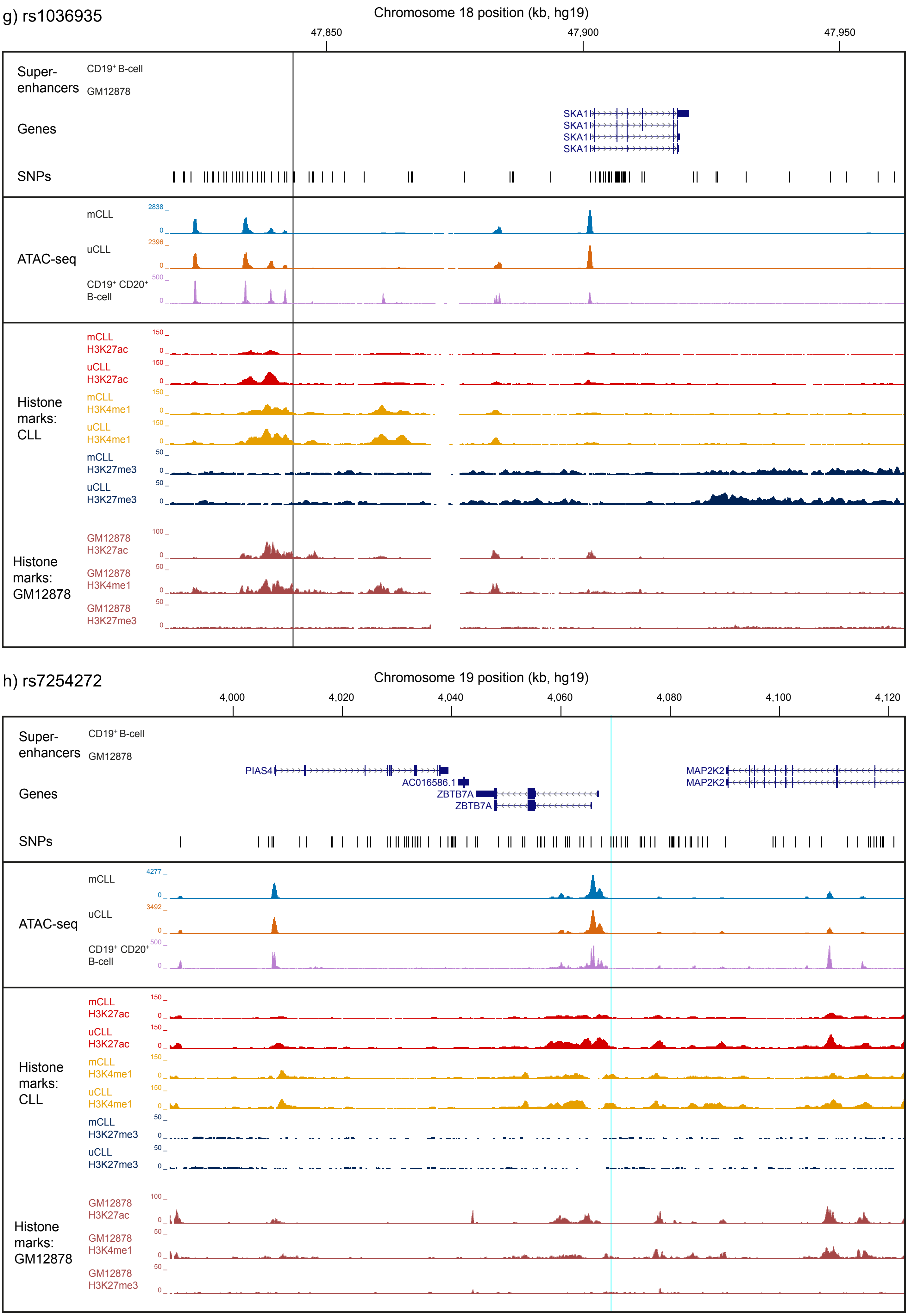

Supplementary Figure 2

i) rs140522

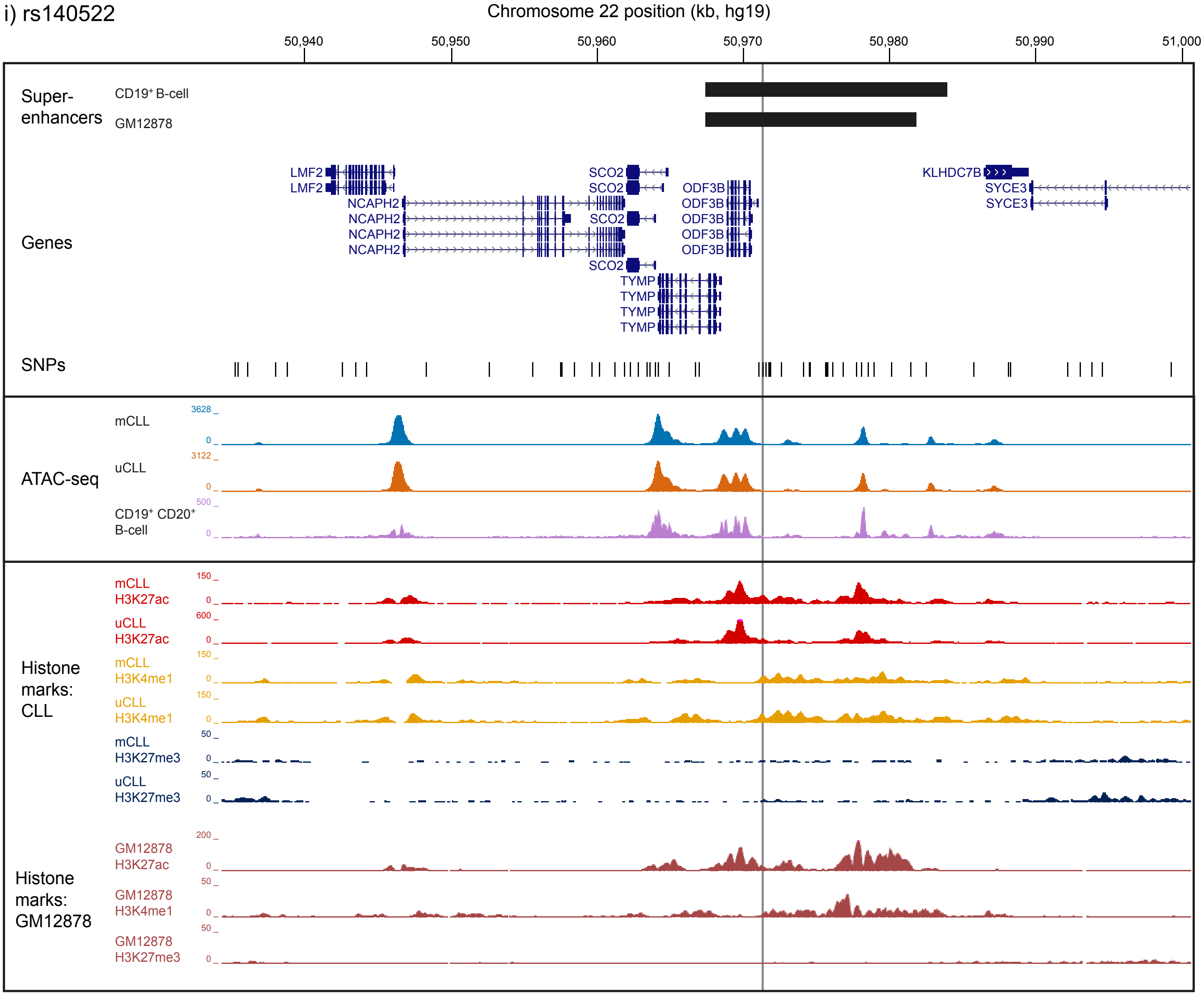

Supplementary Figure 2: Annotation of the regulatory landscape of the nine novel CLL GWAS loci (a-i). Plots illustrate available histone mark ChIP-seq and ATAC-seq data from CLL and B-cell lineage cells. The three boxes in each image are arranged from the top as follows:
Box 1) super-enhancers in CD19$^+$ B-cells and lymphoblastoid cell line GM12878 are indicated by black boxes, coding genes from GENCODE v.24 are labelled in blue, all SNPs (marked by short vertical lines) in linkage disequilibrium $r^2>0.2$ with the sentinel SNP (marked by a vertical line);
Box 2) ATAC-seq peaks derived from *IGHV* mutated (mCLL, blue), unmutated (uCLL, orange) CLL samples plus normal CD19$^+$/CD20$^+$ B-cells; Box 3) Histone mark data from mCLL and uCLL samples for active chromatin marks H3K27ac (red) and H3K4me1 (yellow) plus repressive chromatin mark H3K27me3 (dark blue). Also shown is the same histone mark data for GM12878.

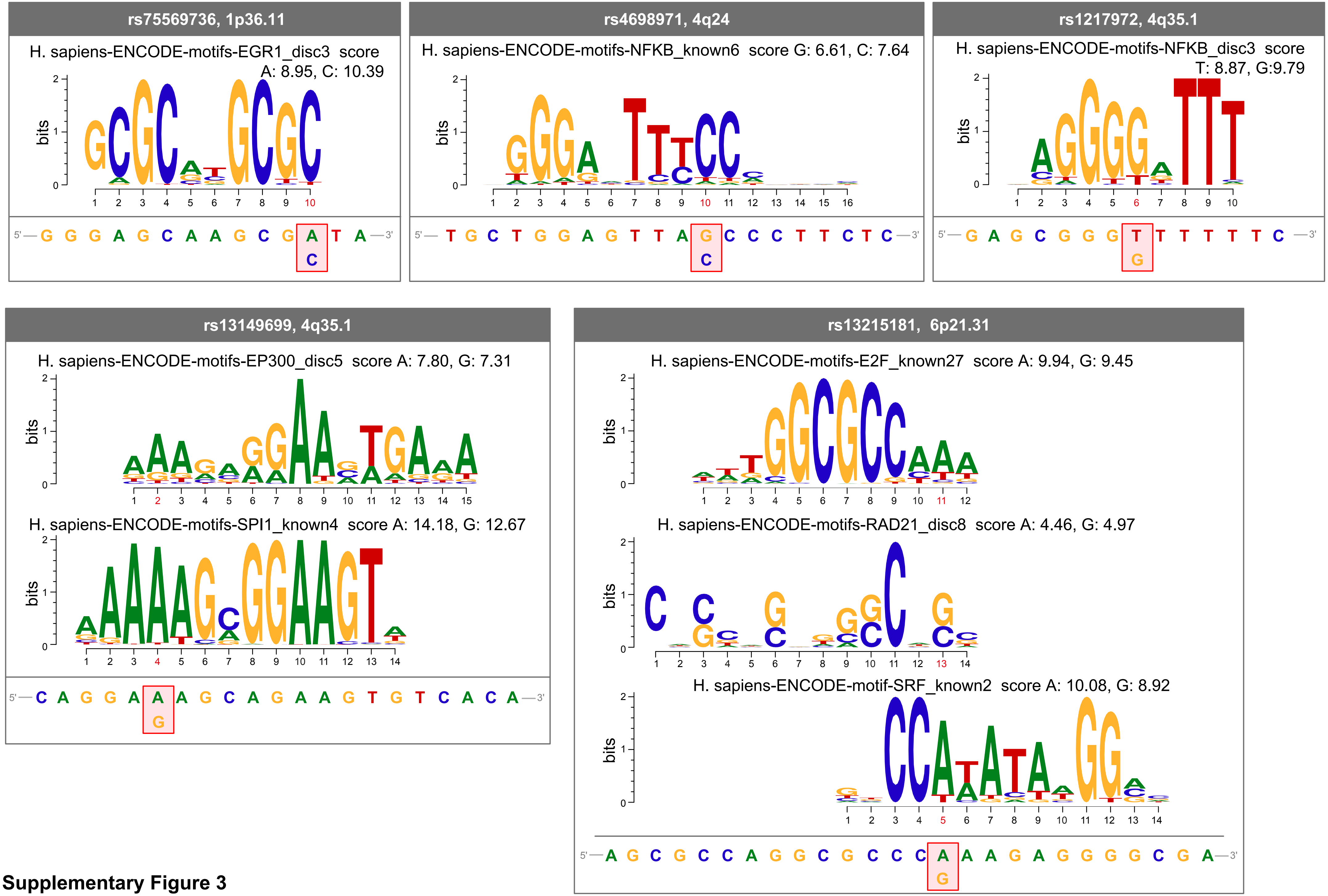

Supplementary Figure 3

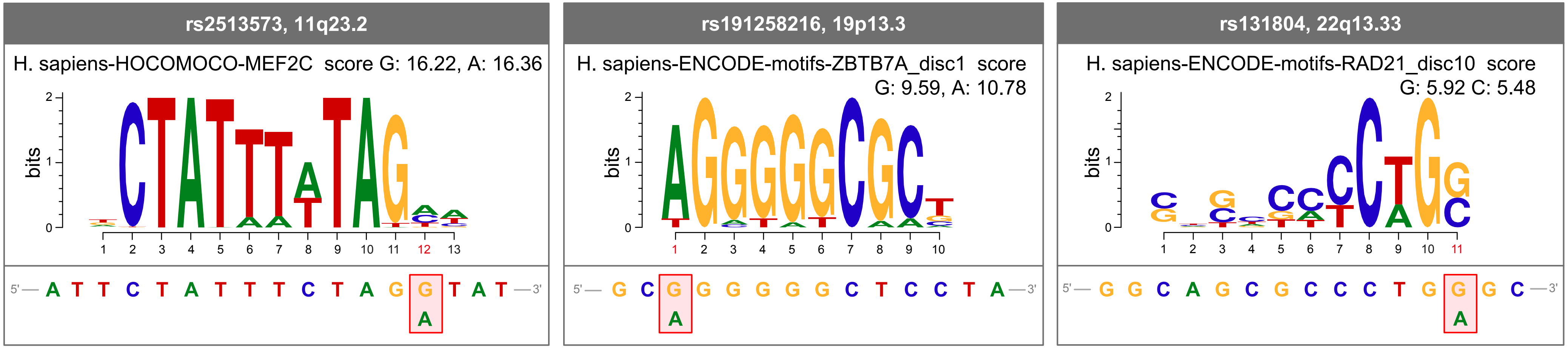

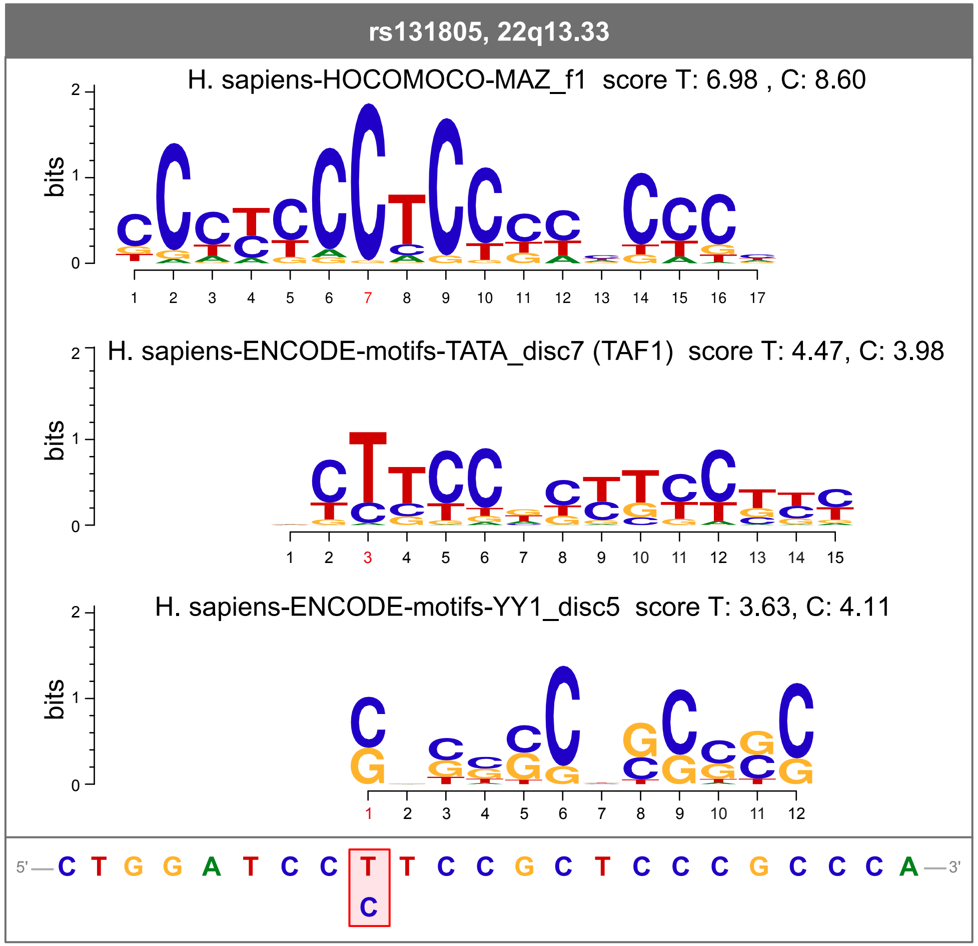

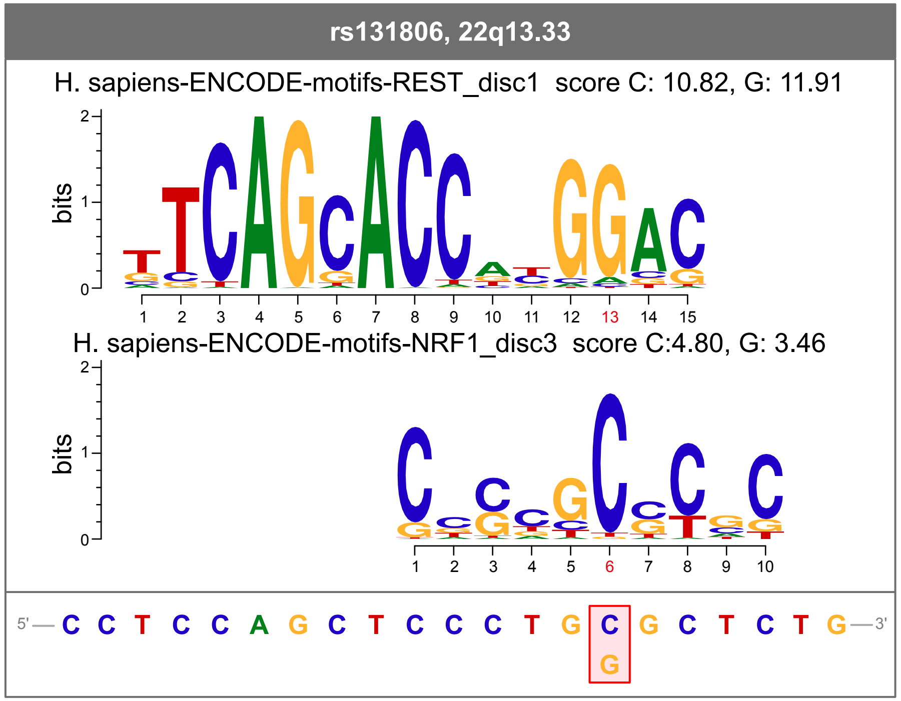

Supplementary Figure 3: Motifs disrupted by SNPs at novel CLL GWAS loci where corresponding protein is also bound in ENCODE ChIP-seq data. Shown are transcription factor binding motifs predicited to by altered by SNPs in LD $r^2>0.2$ with sentinel SNPs at the novel CLL GWAS loci. The transcription factor in question is also bound at the SNP site based on ChIP-seq data from the ENCODE project. Variable nucleotides are highlighted by a pink box. For clarity, where multiple versions of the same motif are disrupted, that with the strongest effect is shown.

Supplementary Table 1: Details of each GWAS and replication dataset

	Subjects		% Male		Mean (SD) age	
Study	Case	Control	Case	Control	Case	Control
**GWAS**						
UK-CLL1	503	2698	70.6	[b]	61.7 (9.9)	[b]
UK-CLL2	1340	2501	64.0	[c]	63.0 (11.2)	[c]
GEC Consortium	387	294	64.4	63.4	61 (17.7)	64 (10.8)
NHL GWAS Consortium						
ATBC	50	236	100	100	72 (6.7)	68 (7.7)
CPS-II	251	220	52.6	49.6	71 (5.9)	68 (6.3)
EPIC	72	265	54.2	45.3	63.5 (8.7)	63 (8.4)
HPFS	19	85	100	100	71 (10)	71 (8.5)
MCCS	57	75	59.7	52.0	65 (10.9)	71 (7.9)
NHS[a]	18	88	0	0	64 (10.3)	64 (7.0)
NYU-WHS[a]	10	53	0	0	70.5 (6.9)	79 (9.3)
PLCO	278	3076	56.5	95.7	70 (6.1)	70 (6.2)
BCCA	26	109	76.9	56.0	65 (9.2)	62 (12.7)
Italian GxE	5	45	80.0	62.2	68 (4.9)	54 (11.8)
NCI-SEER	86	270	59.3	54.1	61.5 (9.6)	59 (12.0)
NSW	13	154	61.5	60.4	64 (10.3)	58 (11.2)
SCALE	395	291	66.3	57.7	63 (8.6)	64 (12.1)
UCSF2	22	10	68.2	60.0	63 (12.2)	44 (15.6)
Yale[a]	39	146	0	0	68 (12.0)	62 (13.5)
ENGELA	44	63	65.9	65.1	62 (9.4)	57 (11.4)
EpiLymph	158	211	65.8	54.0	64 (11.1)	62 (13.0)
Mayo Case-Control	132	1167	71.2	61.2	64 (10.6)	63 (13.4)
MSKCC	36	4	58.3	0	63 (10.8)	39 (10.8)
UCSF	213	747	64.3	57.6	64 (11.2)	62 (13.0)
Utah	324	405	59.5	55.0	63 (22.4)	64 (16.6)
***in silico* replication**						
ICGC	444	609	60.8	59.3	66.0 (12.2)	65.6 (14.1)
WHI[a]	226	228	0	0	NA	NA
***de novo* replication**						
ICR	645	2341	64.9	45.0	64.6 (11.4)	60.3 (10.0)
Mayo	407	1207	67.9	58.0	63 (10.4)	63 (12.0)

[a] Female only study
[b] UK 1958 Birth Cohort (The Wellcome Trust Case Control Consortium)[1]
[c] UK National Blood Service (The Wellcome Trust Case Control Consortium)[1]

Supplementary Table 2: Details of the quality control (QC) filters applied to samples and SNPs for each GWAS

Study	Sample QC[a]						Genotyping QC[b]				
	Cases pre-QC (N)	Controls pre-QC (N)	Exclusion criteria		Cases post-QC (N)	Controls post-QC (N)	Genotyping platform	Exclusion criteria			SNPs passing QC
			Call rate	Other exclusions				MAF	Call rate	HWE-*P*	
UK-CLL1	517	2,698	≤95%	1) Call rate; 2) Sex discrepancy; 3) relatedness	503	2,698	Illumina HumanCNV370-Duo	<0.01	≤95%	$<1x10^{-5}$	301,786
UK-CLL2	1,403	2,501	≤95%	1) Call rate; 2) Sex discrepancy; 3) relatedness; 4) Non-CEU	1,304	2,501	Illumina OmniExpress	<0.01	≤95%	$<1x10^{-5}$	630,366
GEC	396	296	≤95%	1) Non-CEU; 2) PCA outliers	387	294	Affymetrix 6.0	<0.01	≤95%	$<1x10^{-6}$	687,458
Utah	331	420	≤95%	1) Abnormal heterozygosity; 2) Non-CEU; 3) Incomplete phenotype	324	405	Illumina HumanHap 610K	<0.01	≤95%	$<1x10^{-6}$	514,816
UCSF	214	751	≤95%	1) Abnormal heterozygosity; 2) PCA outlier	213	747	Illumina HumanCNV370-Duo	<0.01	≤95%	$<1x10^{-6}$	291,232
NHL GWAS	1,851	6,649	≤93%	1) High missing rate; 2) Sex discrepancy; 3) Non-CEU	1,711	6,568	Illumina OmniExpress	<0.01	≤95%	$<1x10^{-6}$	549,934

[a] Samples were excluded due to call rate (or failed genotyping), ethnicity (principle components analysis or other samples reported to be not of European descent, CEU), relatedness (any individuals found to be duplicated or related within or between data sets through identity-by-state) or sex discrepancy.

[b] Genotypes were excluded due to minor allele frequency (MAF), SNP call rate and departure from Hardy-Weinberg equilibrium (HWE).

Supplementary Table 3: Strongest association signals from previously published risk loci discovered in European populations

Locus	Nearest Gene(s)	Published SNP	Lead SNP in Current GWAS	LD (r^2)	Position (hg19, bp)	Risk Allele	Literature OR (95% CI)	Literature *P*-value	Current GWAS OR (95% CI)	Current GWAS *P*-value	Ref.
2p22.2	*QPCT, PRKD3*	rs3770745			37,596,089	T	1.24 (1.15–1.33)	1.68E-08	1.16 (1.09-1.23)	1.15E-06	2
			rs888096	0.22	37,603,801	A			1.15 (1.09-1.21)	5.20E-08	
2q13	*ACOXL, BCL2L11*	rs13401811			111,616,104	G	1.41 (1.30–1.52)	2.08E-18	1.36 (1.28-1.46)	8.70E-20	2
			rs1002015	0.29	111,616,619	C			1.3 (1.23-1.37)	2.23E-23	
		rs17483466			111,797,458	G	1.39 (1.25-1.53)	2.36E-10	1.37 (1.29-1.45)	2.86E-25	3
			rs58055674	0.55	111,831,793	C			1.41 (1.32-1.5)	2.02E-27	
		rs9308731			111,908,262	A	1.18 (1.12-1.24)	1.69E-10	1.24 (1.18-1.30)	8.93E-17	5
			rs6708784	0.71	111,927,379	G			1.30 (1.24-1.37)	2.67E-25	
2q33.1	*CASP10/CASP8*	rs3769825			202,111,380	A	1.19 (1.12–1.25)	2.50E-09	1.16 (1.1-1.21)	1.31E-08	2
			rs7558911	0.51	202,023,949	A			1.18 (1.12-1.24)	5.05E-11	
2q37.1	*SP110, SP140*	rs13397985			231,091,223	G	1.41 (1.26-1.57)	5.40E-10	1.43 (1.35-1.52)	3.76E-31	3
			rs34004493	0.59	231,154,012	G			1.39 (1.31-1.47)	3.67E-32	
2q37.3	*FARP2*	rs757978			242,371,101	T	1.39 (1.25-1.56)	2.11E-09	1.29 (1.2-1.39)	5.80E-11	4
			rs3755397	0.64	242,294,913	G			1.32 (1.22-1.43)	9.48E-12	
3p24.1	*EOMES*	rs9880772	rs9880772		27,777,779	A	1.18 (1.13-1.25)	9.32E-11	1.16 (1.11-1.22)	1.91E-09	5
3q26.2	*MYNN, TERC*	rs10936599			169,492,101	C	1.26 (1.17-1.35)	1.74E-09	1.19 (1.12-1.26)	6.56E-09	6
			rs1317082	1.00	169,497,585	A			1.19 (1.12-1.26)	5.77E-09	
3q28	*LPP*	rs9815073			188,115,682	C	1.18 (1.11-1.25)	2.28E-08	1.12 (1.06-1.18)	6.75E-05	5
			rs73192661	0.69	188,128,794	C			1.13 (1.07-1.19)	1.73E-06	
4q25	*LEF1*	rs898518			109,016,824	A	1.20 (1.14–1.27)	4.24E-10	1.16 (1.1-1.22)	1.02E-08	2
			rs7690934	0.97	109,025,865	C			1.16 (1.11-1.22)	6.08E-09	
4q26	*CAMK2D*	rs6858698			114,683,844	C	1.31 (1.20-1.44)	3.07E-09	1.08 (1.01-1.16)	2.87E-02	6
			rs1476569	0.39	114,698,696	G			1.14 (1.08-1.2)	4.51E-06	
5p15.33	*TERT*	rs10069690			1,279,790	T	1.20 (1.13-1.26)	1.12E-10	1.19 (1.13-1.26)	1.45E-09	6
			rs7705526	0.27	1,285,974	A			1.18 (1.12-1.25)	5.90E-10	

Locus	Nearest Gene(s)	Published SNP	Lead SNP in Current GWAS	LD (r^2)	Position (hg19, bp)	Risk Allele	Literature OR (95% CI)	Literature *P*-value	Current GWAS OR (95% CI)	Current GWAS *P*-value	Ref.
5p15.33	*CLPTM1L*	rs31490[b]			1,344,458	A	1.18 (1.11-1.26)	1.72E-07	1.09 (1.03-1.14)	1.18E-03	6
			rs10073340	0.25	1,321,873	T			1.13 (1.06-1.20)	2.84E-04	
6p25.3	*IRF4*	rs872071			411,064	G	1.54 (1.41-1.69)	1.91E-20	1.25 (1.19-1.32)	4.22E-19	3
			rs9392504	0.81	412,802	A			1.33 (1.26-1.4)	9.81E-29	
6p25.2	*SERPINB6*	rs73718779	rs73718779	-	2,969,278	T	1.26 (1.16-1.37)	1.21E-08	1.14 (1.06-1.23)	6.98E-04	5
6p21.32	*HLA*	rs674313			32,578,082	T	1.69 (1.41-2.01)	6.92E-09	1.18 (1.12-1.25)	5.74E-09	8
			rs9271176	0.09	32,578,127	G			1.29 (1.22-1.36)	3.16E-20	
6p21.31	*BAK1*	rs210134			33,540,209	G	1.37 (1.22-1.53)	1.03E-12	1.23 (1.17-1.3)	6.48E-14	7
			rs210143	0.82	33,546,930	C			1.26 (1.19-1.33)	5.77E-16	
6q25.2	*IPCEF1*	rs2236256			154,478,440	C	1.23 (1.15-1.30)	1.50E-10	1.14 (1.09-1.2)	1.67E-07	6
			rs4869818	0.97	154,471,225	G			1.15 (1.09-1.21)	4.11E-08	
7q31.33	*POT1*	rs17246404			124,462,661	C	1.22 (1.14-1.31)	3.40E-08	1.15 (1.09-1.22)	5.15E-07	6
			rs2267708	0.25	124,392,512	T			1.16 (1.1-1.22)	8.55E-09	
8q22.3	*ODF1*	rs2511714			103,578,874	G	1.16 (1.11-1.22)	2.90E-09	1.14 (1.08-1.2)	4.33E-07	6
			rs2511713	0.52	103,577,865	G			1.17 (1.1-1.23)	6.04E-08	
8q24.21	*POU5F1B*	rs2456449			128,192,981	G	1.26 (1.17–1.35)	7.84E-10	1.23 (1.17-1.29)	5.76E-15	4
			rs2466029	0.76	128,200,971	G			1.23 (1.17-1.3)	7.47E-16	
9p21.3	*CDKN2B-AS1*	rs1679013			22,206,987	C	1.20 (1.14–1.27)	4.24E-10	1.16 (1.1-1.22)	2.17E-08	2
10q23.31	*ACTA, FAS*	rs4406737			90,759,724	G	1.27 (1.19–1.33)	1.22E-14	1.22 (1.16-1.29)	7.20E-15	2
			rs6586163	0	90,752,018	A			1.23 (1.17-1.29)	1.14E-15	
11p15.5	*C11orf21, TSPAN32*	rs7944004			2,311,152	T	1.20 (1.13–1.27)	2.15E-10	1.09 (1.04-1.15)	4.62E-04	2
			rs2651823	0.51	2,321,650	A			1.18 (1.13-1.25)	5.24E-11	
11q24.1	*SCN3B, GRAMD1B*	rs735665			123,361,397	A	1.45 (1.31-1.61)	3.78E-12	1.63 (1.53-1.72)	4.37E-58	3
			rs35923643	1.00	123,355,391	G			1.63 (1.53-1.72)	4.26E-58	
12q24.13	*OAS3*	rs10735079			113,380,008	G	1.18 (1.12-1.26)	2.34E-08	1.15 (1.09-1.21)	1.05E-07	9
			rs6489882	1.00	113,381,376	G			1.16 (1.1-1.22)	4.76E-08	
15q15.1	*BMF, BUB1B*	rs8024033	rs8024033	-	40,403,657	C	1.22 (1.15–1.30)	2.71E-10	1.26 (1.2-1.32)	7.13E-19	2

Locus	Nearest Gene(s)	Published SNP	Lead SNP in Current GWAS	LD (r^2)	Position (hg19, bp)	Risk Allele	Literature OR (95% CI)	Literature *P*-value	Current GWAS OR (95% CI)	Current GWAS *P*-value	Ref.
15q21.3	*RFX7, NEDD4*	rs7169431			56,340,896	A	1.36 (1.21-1.53)	4.74E-07	1.32 (1.21-1.44)	3.92E-10	[4]
			rs142215530	0.17	56,777,691	G			1.39 (1.29-1.5)	2.46E-18	
15q23	*RPLP1*	rs7176508			70,018,990	A	1.37 (1.26–1.50)	4.54E–12	1.35 (1.28-1.42)	4.55E-31	[3]
			rs11637565	0.96	70,020,525	G			1.35 (1.28-1.42)	1.96E-31	
15q25.2	*CPEB1*	rs783540[b]			83,254,708	G	1.18 (1.10–1.27)	3.67E-06	1.09 (1.04-1.15)	8.83E-04	[4]
			rs17356118		83,237,899	A			1.12 (1.05-1.19)	2.48E-04	
16q24.1	*IRF8*	rs305061[b]			85,975,659	T	1.22 (1.12-1.32)	3.60E-07	1.14 (1.08-1.2)	1.29E-06	[4]
			rs305065		85,973,866	C			1.16 (1.1-1.22)	7.57E-08	
		rs391525			85,944,439	G	1.81 (1.52-2.18)	6.94E-11	1.33 (1.26-1.41)	1.07E-25	[8]
			rs391855	0.62	85,928,621	A			1.34 (1.27-1.41)	1.25E-28	
18q21.32	*PMAIP1*	rs4368253	rs4368253	-	57,622,287	C	1.19 (1.12–1.27)	2.51E-08	1.17 (1.11-1.24)	1.26E-08	[2]
18q21.33	*BCL2*	rs4987855			60,793,549	C	1.47 (1.32–1.61)	2.66E-12	1.36 (1.24-1.49)	3.99E-11	[2]
			rs77551289	0.88	60,788,745	A			1.37 (1.25-1.5)	1.83E-11	
		rs4987852	rs4987852	-	60,793,921	C	1.41 (1.27–1.56)	7.76E-11	1.32 (1.2-1.44)	4.66E-09	
19q13.3	*PRKD2, STRN4*	rs11083846			47,207,654	A	1.35 (1.22–1.49)	3.96E–09	1.24 (1.15-1.34)	2.14E-06	[3]
			rs874460	0.05	47,176,752	C			1.24 (1.15-1.34)	3.37E-08	

[a] Lowest *P*-value variant within a 500kb window in the imputation

[b] Associations that did not reach genome-wide significance in original or current study

Supplementary Table 4: Summary statistics from all datasets for SNPs taken forward to replication phase, and achieved genome-wide significance

		SNP								
		rs34676223	rs41271473	rs71597109	rs57214277	rs3800461	rs61904987	rs1036935	rs7254272[a]	rs140522
	Locus	1p36.11	1q42.13	4q24	4q35.1	6p21.31	11q23.2	18q21.1	19p13.3	22q13.33
	Position (bp, hg19)	23,943,735	228,880,296	102,741,002	185,254,772	34,616,322	113,517,203	47,843,534	4,069,119	50,971,266
	Risk allele	C	G	C	T	C	T	A	A	T
Study	Other allele	A	A	T	C	G	C	G	G	C
UK-CLL1	Case RAF	0.72	0.82	0.70	0.45	0.13	0.16	0.27	0.17	0.37
	Control RAF	0.71	0.78	0.68	0.41	0.12	0.14	0.22	0.17	0.33
	OR	1.06	1.29	1.12	1.16	1.18	1.20	1.30	1.03	1.17
	P-value	0.4868	0.0020	0.1331	0.03	0.1351	0.0622	0.0013	0.7790	0.0251
UK-CLL2	Case RAF	0.74	0.82	0.72	0.43	0.15	0.16	0.24	0.21	0.36
	Control RAF	0.70	0.79	0.68	0.41	0.12	0.13	0.22	0.17	0.32
	OR	1.20	1.23	1.21	1.07	1.31	1.24	1.15	1.28	1.19
	P-value	0.0006	0.0008	0.0002	0.14	0.0001	0.0015	0.0167	0.0001	0.0005
GEC	Case RAF	0.75	0.80	0.72	0.46	0.13	0.13	0.26	0.20	0.36
Consortium	Control RAF	0.71	0.78	0.72	0.40	0.11	0.11	0.21	0.20	0.31
	OR	1.27	1.17	1.00	1.30	1.29	1.20	1.36	1.00[a]	1.22
	P-value	0.1	0.3	1.0	0.03	0.2	0.3	0.031	1.0	0.1
NHL GWAS	Case RAF	0.73	0.81	0.73	0.45	0.14	0.13	0.24	0.20	0.36
Consortium	Control RAF	0.70	0.79	0.69	0.41	0.12	0.11	0.22	0.18	0.33
	OR	1.15	1.16	1.20	1.15	1.15	1.24	1.11	1.17	1.14
	P-value	0.0015	0.0030	0.00002	0.0003	0.0134	0.0003	0.0266	0.0016	0.0016
UCSF	Case RAF	0.75	0.82	0.70	0.42	0.12	0.12	0.25	0.20	0.35
	Control RAF	0.70	0.81	0.69	0.41	0.12	0.11	0.21	0.18	0.31
	OR	1.31	1.11	1.07	1.02	1.01	1.17	1.33	1.11	1.20
	P-value	0.0315	0.4676	0.5954	0.8409	0.9329	0.3461	0.0262	0.4537	0.1120

Utah	Case RAF	0.75	0.81	0.72	0.44	0.13	0.14	0.23	0.21	0.33
	Control RAF	0.74	0.80	0.69	0.40	0.09	0.12	0.21	0.16	0.32
	OR	1.05	1.10	1.17	1.24	1.51	1.22	1.15	1.35	1.07
	P-value	0.6969	0.4670	0.1769	0.0459	0.0141	0.1960	0.2840	0.0296	0.5481
Meta prior	***OR***	***1.16***	***1.19***	***1.17***	***1.14***	***1.21***	***1.23***	***1.17***	***1.18***	***1.16***
to	***95% CI***	***(1.09;1.22)***	***(1.12;1.26)***	***(1.11;1.24)***	***(1.08;1.19)***	***(1.12;1.31)***	***(1.14;1.32)***	***(1.10;1.24)***	***(1.11;1.26)***	***(1.10;1.22)***
replication	***P-value***	***2.69E-07***	***4.69E-08***	***1.02E-08***	***9.56E-07***	***4.20E-07***	***4.44E-08***	***2.81E-07***	***4.61E-07***	***2.20E-08***
ICR	Case RAF	0.76	0.82	0.71	0.42	0.13	0.17	0.24	0.19	0.35
replication	Control RAF	0.70	0.79	0.69	0.42	0.12	0.13	0.22	0.16	0.32
	OR	1.30	1.18	1.12	1.02	1.12	1.38	1.17	1.16	1.14
	P-value	0.0003	0.0426	0.1080	0.7165	0.2321	0.0002	0.0340	0.0696	0.0433
ICGC	Case RAF	0.71	0.84	0.76	0.41	0.10	0.11	0.25	0.21	0.31
replication	Control RAF	0.63	0.80	0.73	0.36	0.09	0.10	0.24	0.20	0.30
	OR	1.47	1.30	1.23	1.24	1.22	1.16	1.08	1.08	1.08
	P-value	0.0001	0.02	0.05	0.0214	0.21	0.31	0.47	0.53	0.42
Mayo	Case RAF	0.73	0.81	0.73	0.43	0.14	0.13	0.23	0.19	0.36
replication	Control RAF	0.70	0.78	0.70	0.40	0.11	0.11	0.22	0.17	0.34
	OR	1.20	1.22	1.20	1.14	1.29	1.27	1.08	1.17	1.08
	P-value	0.0464	0.0514	0.0490	0.1169	0.0417	0.0524	0.4360	0.1226	0.3466
WHI	Case RAF	0.74	0.80	0.71	0.45	0.12	0.12	0.23	0.18	0.37
replication	Control RAF	0.74	0.80	0.72	0.40	0.11	0.13	0.22	0.18	0.36
	OR	1.09	1.03	0.95	1.26	1.05	0.83	1.04	0.94	1.03
	P-value	0.6236	0.8853	0.7724	0.1341	0.8352	0.4112	0.8415	0.7379	0.8696
Meta post	***OR***	***1.19***	***1.19***	***1.17***	***1.13***	***1.20***	***1.24***	***1.15***	***1.17***	***1.15***
replication	***95% CI***	***(1.14; 1.25)***	***(1.13; 1.26)***	***(1.11; 1.22)***	***(1.08; 1.18)***	***(1.13; 1.28)***	***(1.16; 1.32)***	***(1.10; 1.21)***	***(1.10; 1.23)***	***(1.10; 1.20)***
	P-value	***5.04E-13***	***1.06E-10***	***1.37E-10***	***3.69E-08***	***1.97E-08***	***2.46E-11***	***3.27E-08***	***4.67E-08***	***2.70E-09***
	I^2 (P_{het})	***23.54 (0.23)***	***0 (0.95)***	***0 (0.78)***	***0 (0.53)***	***0 (0.69)***	***0 (0.83)***	***0 (0.65)***	***0 (0.55)***	***0 (0.94)***

bp, base pairs; RAF, risk allele frequency; OR, odds ratio derived with respect to risk allele; P_{het}, *P*-value for heterogeneity

[a] GEC data was excluded from the meta-analysis for rs7254272 due to imputation info score <0.8

Supplementary Table 5: Summary statistics from all datasets for SNPs taken forward to replication phase that did not achieve genome-wide significance

		SNP						
		rs736009	rs2071528	rs1289214	rs12654442	rs2731567	rs7026022	rs8181768
	Locus	1p34.2	1q42.3	5q21.2	5q23.2	7p21.1	9q22.33	12q24.22
	Position (bp, hg19)	40,146,417	236,228,681	103,418,704	124,343,851	18,164,761	102,562,216	117,000,196
	Risk allele	T	A	A	T	C	C	A
Study	Other allele	G	G	C	C	G	A	G
UK-CLL1	Cases RAF	0.46	0.20	0.62	0.30	0.04	0.42	0.31
	Controls RAF	0.44	0.18	0.59	0.25	0.04	0.37	0.29
	OR[a]	1.08	1.16	1.11	1.34	1.07	1.27	1.08
	P-value	0.3	0.1	0.1	0.0002	0.7	0.001	0.3300
UK-CLL2	Cases RAF	0.49	0.21	0.63	0.26	0.06	0.40	0.34
	Controls RAF	0.45	0.18	0.58	0.24	0.04	0.37	0.29
	OR	1.17	1.14	1.21	1.12	1.54	1.13	1.25
	P-value	0.001	0.03	0.0001	0.03	0.0001	0.02	0.00002
GEC Consortium	Cases RAF	0.50	0.20	0.63	0.27	0.07	0.39	0.32
	Controls RAF	0.47	0.18	0.61	0.25	0.05	0.36	0.29
	OR	1.14	1.20	1.11	1.13	1.38	1.11	1.13
	P-value	0.001	0.0001	0.009	0.004	0.00004	0.007	0.0033
NHL GWAS Consortium	Cases RAF	0.49	0.21	0.63	0.29	0.06	0.38	0.33
	Controls RAF	0.44	0.16	0.59	0.24	0.04	0.35	0.31
	OR	1.26	1.37	1.18	1.32	1.38	1.09	1.16
	P-value	0.04	0.03	0.16	0.04	0.24	0.49	0.2
UCSF	Cases RAF	0.48	0.19	0.62	0.26	0.07	0.38	0.32
	Controls RAF	0.47	0.18	0.60	0.24	0.05	0.37	0.26
	OR	1.03	1.11	1.12	1.08	1.40	1.05	1.40
	P-value	0.7730	0.4588	0.3137	0.5487	0.1226	0.6415	0.0045

Utah	Cases RAF	0.46	0.19	0.62	0.26	0.06	0.44	0.30
	Controls RAF	0.44	0.19	0.58	0.24	0.06	0.38	0.31
	OR	1.06	1.04	1.11	1.18	0.84	1.28	0.97
	P-value	0.5746	0.7782	0.3321	0.1688	0.4484	0.0208	0.7948
Meta prior to replication	***OR***	***1.14***	***1.17***	***1.14***	***1.16***	***1.34***	***1.14***	***1.16***
	95% CI	***(1.08;1.19)***	***(1.10;1.25)***	***(1.09;1.2)***	***(1.10;1.23)***	***(1.20;1.50)***	***(1.08;1.20)***	***(1.10;1.22)***
	P-value	***4.96E-07***	***6.67E-07***	***2.88E-07***	***1.30E-07***	***1.22E-07***	***3.67E-07***	***6.71E-08***
ICR replication	Cases RAF	0.48	0.20	0.61	0.26	0.04	0.39	0.30
	Controls RAF	0.46	0.19	0.60	0.25	0.05	0.38	0.29
	OR	1.08	1.05	1.06	1.06	0.93	1.06	1.06
	P-value	0.2083	0.5720	0.3684	0.4201	0.6474	0.4146	0.4271
ICGC replication	Cases RAF	0.46	0.20	0.60	0.23	0.07	0.37	0.30
	Controls RAF	0.48	0.22	0.61	0.22	0.07	0.36	0.28
	OR	0.93	0.88	0.94	1.05	1.15	1.02	1.10
	P-value	0.4226	0.2355	0.5206	0.6267	0.4394	0.8711	0.35
Mayo replication	Cases RAF	0.50	0.17	0.61	0.25	0.06	0.40	0.29
	Controls RAF	0.47	0.18	0.62	0.24	0.05	0.36	0.30
	OR	1.15	0.98	0.99	1.05	1.29	1.21	0.97
	P-value	0.0762	0.8691	0.9198	0.6204	0.1407	0.0248	0.7328
WHI replication	Cases RAF	0.47	0.19	0.59	0.24	0.06	0.36	0.31
	Controls RAF	0.45	0.21	0.55	0.24	0.06	0.33	0.30
	OR	1.20	0.96	1.16	0.95	1.32	1.15	1.02
	P-value	0.2391	0.8164	0.3540	0.7718	0.3885	0.3866	0.8962
Meta post replication	***OR***	***1.12***	***1.12***	***1.11***	***1.13***	***1.28***	***1.13***	***1.13***
	95% CI	***(1.07; 1.17)***	***(1.06; 1.18)***	***(1.06; 1.16)***	***(1.08; 1.19)***	***(1.17; 1.40)***	***(1.08; 1.18)***	***(1.08; 1.18)***
	P-value	***2.25E-07***	***4.64E-05***	***3.42E-06***	***5.27E-07***	***1.48E-07***	***7.00E-08***	***4.12E-07***
	I^2 (P_{het})	***0 (0.51)***	***28.30 (0.18)***	***1.95 (0.42)***	***0.55 (0.43)***	***31.89 (0.15)***	***0 (0.55)***	***33.64 (0.14)***

bp, base pairs; RAF, risk allele frequency; OR, odds ratio derived with respect to the risk allele; P_{het}, *P*-value for heterogeneity

Supplementary Table 6: Concordance between imputed and directly typed SNP genotypes in a subset of UK and US CLL cases

		UK-CLL2 cases					Mayo CLL cases				
			Direct genotyping					Direct genotyping			
SNP	Genotype	Imputation	Concordant	Discordant	No data	r^{2} [a]	Imputation	Concordant	Discordant	No data	r^{2} [a]
	AA	165	161	0	4		123	123	0	0	
rs34673223	Aa	114	109	1	4	0.9954	92	90	2	0	0.9522
	aa	18	18	0	0		15	16	1	0	
	AA	200	187	2	11		144	144	0	0	
rs41271473	Aa	84	80	0	4	0.9889	71	71	0	0	1.0000
	aa	13	12	0	1		15	15	0	0	
	AA	162	155	0	7		121	117	4	0	
rs71597109	Aa	110	108	0	2	0.9959	92	93	1	0	0.9957
	aa	25	24	1	0		17	17	0	0	
	AA	108	100	5	3		73	74	1	0	
rs57214277	Aa	148	147	1	0	0.9665	115	111	4	0	0.9696
	aa	41	41	0	0		42	45	3	0	
	AA	217	213	1	3		165	165	0	0	
rs3800461	Aa	73	71	1	1	0.9866	58	58	0	0	0.9826
	aa	7	7	0	0		7	7	0	0	
	AA	220	211	2	7		171	169	2	0	
rs61904987	Aa	60	55	2	3	0.9715	54	54	0	0	0.9957
	aa	17	13	1	3		5	5	0	0	
	AA						131	130	1	0	
rs1036935	Aa	Directly genotyped in discovery dataset					82	82	0	0	0.9913
	aa						17	18	1	0	
	AA						148	156	8	0	
rs7254272	Aa	Directly genotyped in discovery dataset					76	64	8	0	0.8609
	aa						6	10	4	0	

		UK-CLL2 cases					Mayo CLL cases				
			Direct genotyping					Direct genotyping			
SNP	Genotype	Imputation	Concordant	Discordant	No data	r^{2} [a]	Imputation	Concordant	Discordant	No data	r^{2} [a]
rs140522	AA	Directly genotyped in discovery dataset					94	94	0	0	0.9696
	Aa						109	108	1	0	
	aa						27	28	1	0	
rs736009	AA	85	83	1	1	0.9798	61	61	0	0	1.0000
	Aa	144	138	2	4		121	121	0	0	
	aa	68	64	0	4		48	48	0	0	
rs2071528	AA	194	185	0	9	0.9938	160	161	1	0	0.9957
	Aa	96	90	1	5		62	61	1	0	
	aa	7	7	0	0		8	8	0	0	
rs1289214	AA	123	120	1	2	0.9962	92	93	1	0	1.0000
	Aa	138	138	0	0		113	112	1	0	
	aa	36	36	0	0		25	25	0	0	
rs12654442	AA	146	144	0	2	0.9955	113	112	1	0	1.0000
	Aa	132	131	1	0		94	95	1	0	
	aa	19	19	0	0		23	23	0	0	
rs2731567	AA	256	254	0	2	1.0000	210	211	1	0	0.9957
	Aa	41	41	0	0		20	19	1	0	
	aa	0	0	0	0		0	0	0	0	
rs7026022	AA	Directly genotyped in discovery dataset					86	86	0	0	1.0000
	Aa						109	109	0	0	
	aa						35	35	0	0	
rs8181768	AA	122	119	1	2	0.9870	94	94	0	0	1.0000
	Aa	150	142	2	6		110	110	0	0	
	aa	25	24	0	1		26	26	0	0	

[a] r^{2} indicates Pearson product-moment correlation coefficient between imputed and sequenced genotype.

Supplementary Table 7: Relationship between genome-wide significant SNPs genotype and patient outcome (overall survival)

		UK				Mayo				Mayo Replication	
SNP	Alleles[a]	HR (95% CI)	*P*	Adj HR (95% CI)[b]	Adj *P*	HR (95% CI)	*P*	Adj HR (95% CI)[c]	Adj *P*	HR (95% CI)	*P*
rs34676223	C/A	1.04 (0.86-1.25)	0.71	1.14 (0.93-1.40)	0.21	0.92 (0.65-1.28)	0.61	0.83 (0.54-1.25)	0.37	0.97 (0.75-1.28)	0.85
rs41271473	G/A	0.81 (0.67-0.99)	0.04	0.89 (0.71-1.10)	0.27	1.39 (1.00-1.95)	0.05	1.38 (0.89-2.13)	0.14	1.1 (0.83-1.46)	0.52
rs71597109	C/T	0.88 (0.73-1.10)	0.21	0.83 (0.67-1.02)	0.08	0.91 (0.65-1.28)	0.60	0.81 (0.54-1.22)	0.31	1.07 (0.83-1.40)	0.6
rs57214277	C/T	1.08 (0.91-1.28)	0.41	1.01 (0.83-1.22)	0.94	1.19 (0.88-1.63)	0.26	1.05 (0.71-1.55)	0.81	1.1 (0.86-1.39)	0.46
rs3800461	G/C	0.97 (0.76-1.24)	0.82	1.08 (0.83-1.40)	0.57	1.14 (0.77-1.70)	0.51	1.12 (0.71-1.78)	0.63	1.04 (0.74-1.44)	0.84
rs61904987	C/T	0.88 (0.70-1.10)	0.26	0.88 (0.68-1.13)	0.32	0.81 (0.51-1.29)	0.37	0.72 (0.39-1.32)	0.29	1.16 (0.84-1.60)	0.38
rs1036935	G/A	1.01 (0.84-1.21)	0.92	0.88 (0.68-1.13)	0.32	1.14 (0.81-1.59)	0.46	1.01 (0.67-1.52)	0.98	0.96 (0.74-1.26)	0.77
rs7254272	G/A	0.99 (0.79-1.24)	0.94	0.92 (0.71-1.18)	0.5	0.86 (0.58-1.27)	0.44	0.79 (0.48-1.30)	0.35	0.86 (0.64-1.17)	0.33
rs140522	C/T	0.96 (0.81-1.14)	0.63	1.11 (0.75-1.08)	0.27	1.13 (0.83-1.53)	0.44	1.2 (0.82-1.75)	0.34	0.93 (0.73-1.18)	0.53

HR, hazard ratio

[a] Major allele/minor allele. Hazard ratios are calculated with respect to the minor allele

[b] Adjustment for age, Binet stage at diagnosis and IGHV mutation status

[c] Adjustment for age, Rai stage at diagnosis and IGHV mutation status

Supplementary Table 8: Transcription factor binding and motif annotation for 47 SNPs overlapping CLL ATAC-seq peaks

SNP	Locus	Position	LD[a]	GERP[b]	Bound proteins (ENCODE ChIP-seq – all cells)[c]	Altered motifs from motifbreakR[d]
rs2813876	1p36.11	23945914	0.29	2.60	EZH2, HDAC1, RCOR1, ZNF143, HDAC2, **POLR2A**, **CTCF**	NHLH1, NR2E1, NR2F6, RARA
rs75569736	1p36.11	23946020	0.37	4.56	EZH2, HDAC1, RCOR1, ZNF143, HDAC2, **POLR2A**, **CTCF**, **EGR1**, **BCL3**, E2F6, HMGN3, MYC, **FOXM1**, **MAZ**	**EGR1, GATA1**, NRF1
rs72663260	1p36.11	23946443	0.37	4.22	EZH2, **POLR2A**, HMGN3, **FOXM1**, **MAZ**, CTBP2, **EP300**, **RELA**, **TCF12**, **TAF1**, E2F1, **MEF2A**, **ELF1**, **NFYB**, TBP, MAX, **ZEB1**	GRHL1, MYB, NR1H, RFX5, SCRT1, SCRT2, TFCP2
rs12136157	1q42.13	228703236	0.26	0.20	PML, MYC, MAZ, **CTCF**, **SMC3**, RFX5, RCOR1, **RAD21**	ARID3A, CPEB1, HOXC12, HOXC13, HOXD10, HOXD11, IRF1, PRDM1, SOX10, SOX8, T1ISRE
rs55724370	1q42.13	228782630	0.30	0.14	**BHLHE40**, POLR2A, CHD2, TBP, BRCA1	JUND, CACBP, GTF2I, IRF5, NR0B1, PAX5, UA7, WT1
rs7683892	4q24	102712542	0.36	-2.65	TCF7L2, TCF12, EZH2	GFY, PAX3, PURA, UA8, HIVEP1, ZFX
rs4414957	4q24	102723246	0.42	0.23	**SPI1**	MTF1, THRB
rs4610336	4q24	102772299	0.37	0.43		POU5F1, FOS, HNF4A, IRF5, MTF1, NR2F6, POU2F1, POU2F2, POU, RXRA, TBR1
rs4698971	4q24	102772861	0.47	-0.17	**RELA**	BCL3, MZF1, **NFKB (RELA)**, HIVEP2
rs199914135	4q24	102777542-5	0.45	-1.80		
rs34210876	4q24	102777543-6	0.5	-1.43		

SNP	Locus	Position	LD[a]	GERP[b]	Bound proteins (ENCODE ChIP-seq – all cells)[c]	Altered motifs from motifbreakR[d]
rs11933157	4q24	102792348	0.48	2.11		AIRE, FOS, CDX1, CDX2, FOXC1, FOXF1, FOXI1, FOXJ2, FOXJ3, FOXL1, FOXO1, FOXO4, FOXP1, FOXP2, FOXQ1, HOXA11, HOXA13, HOXB13, HOXC13, HOXD13, IRX2, IRX5, DMC1, NANOG, SOX2-OCT4, POU5F1, SOX2, TBP, ZNF143
rs2216546	4q24	102793500	0.51	-6.06		EP300, MECOM, FOXA1, FOXO1, FOXO3, FOXO4, GATA, HDAC2, IRF3, IRF4, IRF5, IRF7, OBOX5, RUNX1, T1ISRE, TEF, TRIM28
rs2850375	4q24	102893970	0.24	-1.12		ARNT, CDX1, HIF1A, HIF2A, HOXA10, ZSCAN4
rs2850378	4q24	102915967	0.23	2.01		CDX1, CDX2, GFI1B, HOXA10, HOXA11, HOXA13, HOXC10, HOXC11, HOXC12, HOXD13, HOXD9, SREBF1, JUN
rs62322738	4q24	102929034	0.21	0.07	**ATF2**	JUN, HDAC2, HMGA2, LHX2, MEOX2, POU1F1, POU2F3, POU3F3, POU4F2, UA10
rs12510921	4q35.1	185189278	0.35	1.12	RBBP5, **YY1**, **POLR2A**, E2F6, **MAX**, MYC, **NFATC1**, **IKZF1**, **RELA**, **CEBPB**, **BCLAF1**, IRF1, **MXI1**, **RUNX3**, **CHD2**, **TAF1**, SIN3AK20, **TBP**, **NFIC**, **EBF1**, STAT1, STAT2, **EP300**, **ATF2**, **BHLHE40**, **IRF4**, **FOXM1**, **SRF**, **POU2F2**, **BATF**, **MEF2C**, **PML**, **MTA3**, **EGR1**, **MEF2A**, **PAX5**, **SPI1**, **RAD21, SMC3, BCL11A, ELF1**	BCLAF1, MAFB

SNP	Locus	Position	LD[a]	GERP[b]	Bound proteins (ENCODE ChIP-seq – all cells)[c]	Altered motifs from motifbreakR[d]
rs1217971	4q35.1	185209881	0.23	-0.83	**POLR2A, MTA3, NFATC1, RUNX3**, **BCLAF1, RELA, NFIC, BCL3, POU2F2**, **CEBPB, PML, MAZ, EP300, MEF2C, FOXM1, ATF2, IKZF1, RAD21, SMC3, SPI1, SP1, BATF, BCL11A, CHD2, BHLHE40**, **STAT3, TBL1XR1, MEF2A, MAX, MXI1, WRNIP1, TBP, IRF4, RCOR1, CHD1**	AIRE
rs1217972	4q35.1	185210039	0.23	1.27	**POLR2A, NFATC1, RELA, NFIC, POU2F2, CEBPB, PML, MAZ, EP300, FOXM1, IKZF1, CHD2, BHLHE40, STAT3, TBL1XR1, MAX, MXI1, WRNIP1, TBP, RCOR1, CHD1, TAF1, EBF1, PAX5, YY1,** ESR1, RAD21	**NFKB (RELA)**, PRDM1, PTF1A, REL, RELB, RREB1, ZBTB33
rs793892	4q35.1	185249147	0.32	-2.03	**POLR2A, CHD1, TBP, WRNIP1, MTA3**	CTCF, E2F3, E2F4, E2F2, IRX2, IRX3, NR2E1, RAD21
rs13149699	4q35.1	185251371	0.83	2.43	**POLR2A, TBP, RELA, TAF1, RUNX3, MXI1, CEBPB, NFYB, IRF4, TBL1XR1, NFIC, ATF2, EBF1, SMC3, ELF1, PAX5, MAX, CHD2, EP300, YY1, MEF2A, POU2F2, RAD21, MTA3, MEF2C, PML, FOXM1, NFATC1, EGR1, CTCF, BHLHE40, SP1**, FOXA2, **BCL11A, USF2, USF1**, FOXA1, **SPI1, TCF3, TCF12**	**EP300**, GATA2, **SPI1**, SPIB, SPIC
rs793899	4q35.1	185252818	0.92	-2.71	EP300, GATA3, ESR1	BHLHE41, TCF4, LMO2, MYOD1, NRF1, TCF3, TFE3, ZEB1
rs793900	4q35.1	185253019	0.92	-1.45	ESR1	E2F2, HOXA13, HOXB13, HOXC13, HOXD13, MYEF2, NR2E1, NR4A2
rs3800456	6p21.31	34664207	0.45	1.12	RBBP5, MAX, **CHD1**, SAP30, **SIN3A**, ELF1, **POLR2A**, HDAC2, **TAF1**, UBTF, PHF8, SIN3AK20	EGR1, EGR3, IRF1, SP1, TBP, YY1, ZNF143
rs9469860	6p21.31	34724815	0.67	-1.58	CBX3, **CTCF, POLR2A, RUNX3**, BCL3, TCF12, **PML,** MAX, **NFIC**, MYC, **USF2**, **USF1**, BHLHE40, ATF3, TEAD4, **ATF2**, REST	CEBP, EBF1, PAX5, RFX2, SOX4, TLX1

SNP	Locus	Position	LD[a]	GERP[b]	Bound proteins (ENCODE ChIP-seq – all cells)[c]	Altered motifs from motifbreakR[d]
rs111498384	6p21.31	34759503-6	0.69	0.69	**CTCF, POLR2A**, CHD1, BHLHE40, NR2F2, CBX3, TEAD4, NFIC, PHF8, RBBP5, SIN3A, E2F1, PML, KAP1, GATA1	
rs13215181	6p21.31	34759881	0.25	-2.35	**CTCF, POLR2A**, PHF8, RBBP5, SIN3A, E2F1, PML, KAP1, GATA1, KDM5B, TFAP2C, HMGN3, IRF1, **TAF1**, GABPA, MAX, ARID3A, MXI1, E2F4, MAZ, TBP, **YY1, ELF1**, SIN3AK20, CEBPD, MYC, GTF2B, **RAD21, SRF**, GRp20, **PAX5, EGR1, TCF3**, ZBTB7A, CCNT2, CHD1, ETS1, SMARCB1	**E2F1**, **RAD21**, SMC3, **SRF**
rs6934662	6p21.31	34759884	0.25	1.76	**CTCF, POLR2A**, PHF8, RBBP5, SIN3A, E2F1, PML, KAP1, GATA1, KDM5B, TFAP2C, HMGN3, IRF1, **TAF1**, GABPA, MAX, ARID3A, MXI1, E2F4, MAZ, TBP, **YY1**, **ELF1**, SIN3AK20, CEBPD, MYC, GTF2B, **RAD21, SRF**, GRp20, **PAX5, EGR1, TCF3**, ZBTB7A, CCNT2, CHD1, ETS1, SMARCB1	
rs7769961	6p21.31	34760162	0.22	1.65	**POLR2A**, PHF8, RBBP5, E2F1, HMGN3, SIN3AK20, CCNT2, CHD1, ETS1, SMARCB1, NR2F2, E2F6	KLF15, NR0B1, SMAD1, SMAD3, ZNF143
rs7769820	6p21.31	34760228	0.22	-5.72	**POLR2A**, PHF8, RBBP5, HMGN3, SIN3AK20, CCNT2, CHD1, SMARCB1, NR2F2, E2F6	BCLAF1, EGR1, ELF1, GLIS2, GLIS3, MAZ, PLAG1, POU2F2, SETDB1, SIN3A, SRF, TAF1, UA5, WT1, ZBTB7B, ZNF219, ZNF143, ZNF281
rs7752060	6p21.31	34760325	0.21	3.59	**POLR2A**, PHF8, RBBP5, CHD1, SMARCB1, NR2F2, E2F6, ZNF263	CACD,DMRTC2,KLF1,KLF4,KLF7,MAZ,PATZ1,PPARA,RFX4,RREB1,SP1,SP3,ZBTB7B,ZNF219
rs2513573	11q23.2	113718477	0.47	-0.16	**MEF2C, MEF2A**	BCL6B, **MEF2C**, POU2F2, TBP
rs746263	18q21.1	47834577	0.75	0.92	**POLR2A**, JUND, STAT3, ELF1, NR3C1	CACD, KLF1, EP300, TOPORS, ZNF628, ZSCAN4

SNP	Locus	Position	LD[a]	GERP[b]	Bound proteins (ENCODE ChIP-seq – all cells)[c]	Altered motifs from motifbreakR[d]
rs9949024	18q21.1	47839240	0.99	0.24	**BCLAF1, NFIC, IRF4, POU2F2, RELA, SP1**, **EP300, BATF, EBF1, MTA3, PAX5, RUNX3, SPI1, MEF2C, FOXM1, NFATC1, BCL11A, MEF2A**	BARHL2, BARX1, EN1, GBX2, HERPUD1, HESX1, HMX1, HMX3, HOXA5, HOXB6, ISX, LHX9, MAFK, MSX1, MSX2, NOBOX, PRDM9, RFX2, RFX3, RFX4, RHOXF1, SOX10, SOX8, SOX9
rs1373184	18q21.1	47841892	1	-0.47	**PML, MTA3, STAT5A, RELA, NFATC1, FOXM1, CEBPB, TBL1XR1, NFYB, EP300, BCL3, NFIC, RUNX3, PAX5, POU2F2, IRF4, MEF2A, EBF1, SP1, MEF2C, BCL11A, RAD21**	ETV7, HBP1, NKX3-1, NR2E1, NR2F6, POU4F1, POU4F2, POU4F3, ZFHX3
rs3862689	18q21.1	47847254	0.96	0.16	**FOXM1, NFIC, POU2F2, TBP, EP300, MEF2A, MEF2C, RUNX3**	FOXP3, HSF2, POU2F1, SIX1
rs7504604	18q21.1	47847364	0.97	0.16	**FOXM1, NFIC, POU2F2, TBP, EP300, MEF2A, MEF2C, RUNX3**	NKX2-1, NKX2-6, CBFB, RUNX1, RUNX2, SRF, UA11
rs11663953	18q21.1	47901471	0.27	1.47	**PML**, RBBP5, **MTA3, POLR2A, GABPA, TAF1**, MYC, **RELA**, KDM5B, *TBP, PAX5*, HMGN3, **MAX, MAZ, YY1**, REST, CCNT2, JUND, **CHD2**, FOS, **ELF1**, E2F6, **ELK1**, **SIN3A**, **FOXM1**, CEBPD, IRF1, **SP1**, BRCA1, **E2F4, TCF12**, CEBPB, FOXP2, **MXI1, IRF4**, ATF3, THAP1, CREB1, ETS1, **PBX3, NRF1, ATF2**, SIN3AK20, MAFK, MAFF, PHF8, HDAC1, SAP30	ZSCAN16
rs735842	19p13.3	4007080	0.28	-4.06	CHD1, RBBP5, **POLR2A**	CACD, CTCF, EBF1, KLF1, FOXO4, FOXO6, KLF3, KLF4, NR0B1, RBPJ, ZNF740, ZNF75A

SNP	Locus	Position	LD[a]	GERP[b]	Bound proteins (ENCODE ChIP-seq – all cells)[c]	Altered motifs from motifbreakR[d]
rs58300298	19p13.3	4058447	0.45	1.49	POLR2A, SMARCC1, TFAP2C, CBX3, CCNT2, TFAP2A, HDAC2, RCOR1, JUNB, GTF2F1, MAZ, ZNF143, JUN, JUND, SIN3AK20, STAT3, E2F4, ZBTB7A, **CTCF**, CHD2, MAX, FOS, UBTF, EP300, CEBPB, **EGR1**, FOSL2, TAF1, SP1, TEAD4, MXI1, BRCA1, FOSL1, SMC3, **EBF1**, YY1, ATF3, TBP, RAD21	
rs191258216	19p13.3	4065415	0.26	1.51	**POLR2A**, RBBP5, ZBTB7A, **SIN3A**, FOSL1, **TAF1, TCF3**	CHD2, CTCFL, EGR1, EGR2, EGR3, EGR4, GLIS2, GLIS3, HDAC2, INSM1, NRF1, RAD21, SMC3, SRF, ZBTB33, **ZBTB7A**, ZIC1, ZIC3, ZNF219
rs188955288	19p13.3	4067314	0.82	1.63	**POLR2A**, ZBTB7A, GATA1, HMGN3, MAX, TAL1, MYC, TAF1, **EGR1, EBF1**, KDM5B, PHF8	ATF4, EGR3, EGR4, MTF1, NR3C1, SP4, ZBTB7B, ZNF143
rs131806	22q13.33	50963965	0.66	1.54	**POLR2A, STAT5A, NFIC**, MYBL2, **MYC, MAX**, BCL3, **PML**, SMARCB1, **TAF1**, E2F6, KDM5B, REST, TFAP2C, PHF8, **MXI1**, E2F1, IRF1, **MAZ**, CCNT2, RBBP5, ZKSCAN1, **RELA**, ELK1, **NRF1**, MAFK, **POU2F2**, **ZNF143, NFATC1**, CREB1, **SIN3A, ATF3**, **BHLHE40**, GABPA, **USF2, SP1**, JUN, **CHD2**	HNF4, HNF4G, MTF1, NR3C1, **NRF1**, **REST**, ZNF589
rs131805	22q13.33	50964153	0.55	-2.83	**POLR2A, MYC, MAX, PML**, SMARCB1, **TAF1**, E2F6, REST, TFAP2C, **MXI1**, E2F1, IRF1, **MAZ**, CCNT2, RBBP5, ZKSCAN1, **RELA**, ELK1, **NRF1**, MAFK, **POU2F2**, **ZNF143**, **NFATC1**, CREB1, **SIN3A, ATF3, BHLHE40**, GABPA, **USF2, SP1**, JUN, **CHD2**, **USF1**, TBP, SIN3AK20, UBTF, **ELF1, E2F4,** TCF7L2, FOXP2, **EGR1, YY1**, EP300, CHD1, **CTCF, PBX3, PAX5, RAD21**, MAFF, **RUNX3**, SMC3, PHF8	POLR3A, ETV6, **MAZ**, NFE2L2, SP4, SPI1, **TAF1**, **YY1**

SNP	Locus	Position	LD[a]	GERP[b]	Bound proteins (ENCODE ChIP-seq – all cells)[c]	Altered motifs from motifbreakR[d]
rs131804	22q13.33	50964862	0.66	-2.78	**POLR2A, MYC, TAF1**, ZNF263, **TBP**, FOXP2, **ELF1, RELA**, PHF8, **MAZ, MAX**, CCNT2, **MXI1, BCL3, MEF2C, CTCF**, E2F6, **YY1**, HDAC2, **MTA3**, BACH1, **RAD21, ZNF143**, SMC3, **RUNX3**, ZBTB7A, CTCFL	**RAD21**, REST, ZFX
rs470118	22q13.33	50977738	0.32	-0.42	TFAP2C, **POLR2A, MYC**, TFAP2A, **MAX**, E2F6, **MXI1, USF1, TBL1XR1**, FOXP2, **USF2, CHD1, BHLHE40, E2F4, WRNIP1, CHD2, POU2F2, SMC3, NFATC1**	BCLAF1, CTCF, EP300, GFY, KLF8, REST, SIN3A
rs140521	22q13.33	50978024	0.34	0.76	**POLR2A, MYC**, TFAP2A, E2F6, **CHD1, NFATC1, NFYB, PML, MTA3**, KAP1, **FOXM1, TAF1**, TCF12, MAFK, STAT1, STAT2, IRF1, **RUNX3, EP300, TBP**, JUND, **SP1, RELA, BCL11A**, STAT3	ESRRA, LHX8, PDX1, POU2F1, RARA, ZEB1

LD, linkage disequilibrium; GERP, genomic evolutionary profiling rate

[a] LD (r^2) with top SNP at locus

[b] GERP score above +2 is considered indicative of an evolutionarily constrained site.

[c] All proteins bound at the SNP site based on ChIP-seq data from the ENCODE project. Proteins bound in lymphoblastoid cell lines are emboldened

[d] Transcription factor binding motifs predicted to be altered by the SNP. Motifs that match a protein bound in ChIP-seq data are emboldened

Supplementary Table 9: Primers used for replication genotyping. a) KASPar primers[a]; b) Sequenom primers

a)

SNP	Allele 1 primer[a]	Allele 2 primer	Common primer
rs34676223	CAAAACAAAAAGAAAAACGATACTACAAACG	AACAAAACAAAAAGAAAAACGATACTACAAACT	GTTTGACTCACCCTTGAAACTCTAAGATA
rs736009	CACTGGGTTGCTATCCTCTTGC	CCACTGGGTTGCTATCCTCTTGA	GTGGTTGCAAACCAGTGGTTGCTAT
rs41271473	GGATTGAGGCAGTTTGGTGCAC	AGGATTGAGGCAGTTTGGTGCAT	TGTGGAGATCAGAAAGTGACATTTGCTTT
rs2071528	CCCGCGCTCTGCTCCCC	CCCGCGCTCTGCTCCCT	CCGGTGCCTGCGATTCTCGAAA
rs71597109	AGGACACCAGCTGGATTTCG	AGGACACCAGCTGGATTTCA	GCGAAGGGAACATCAGAACACTCTA
rs57214277	CCTTTTGCCTTCCACATGACCG	CCTTTTGCCTTCCACATGACCA	GGAAGCGTGGCAGCTTCTGCTT
rs1289214	ATTATTTGCATATCAGTTGTCATGTGTTAG	GAATTATTTGCATATCAGTTGTCATGTGTTAT	GAAGATGAGAGCCAGCGTATATTTCAAAT
rs12654442	ATACTCCCATTGTGACAGGTAC	ATACTCCCATTGTGACAGGTAT	CAGAAAAGAATCTAACAGTCTAGTGAGAAA
rs3800461	CCTCCAGCAGTGGTTGCCC	CCTCCAGCAGTGGTTGCCG	GCCACTATGTTTTAGCATACCTTCTTCTA
rs2731567	GGTGTGTTGGAGGAAGTTGACTC	GGTGTGTTGGAGGAAGTTGACTG	ACCCTCATTCCCCAGGACTGCT
rs7026022	CCAATAAAAAAGGACCTCCAAATAAAAAAGTA	CAATAAAAAAGGACCTCCAAATAAAAAAGTC	CTCTAGTCACAGGGGTCTGGTTAT
rs61904987	CCAGCCTAGAGACGGTGATCG	CCAGCCTAGAGACGGTGATCA	CAGATGCCAGTCAAAGCAGCTCTT
rs8181768	AGGCATCCTGACTCATGCAGTG	AAAGGCATCCTGACTCATGCAGTA	AAGAGGTCTACCTCCTGCTCAGATT
rs1036935	GTAGTTTTAAAAAATGGCCTCAAACTCTTT	AGTTTTAAAAAATGGCCTCAAACTCTTC	TATACTCAGTGGTCAAAGCAGTCAGAAA
rs7254272	GGCACGATGGTTCATTCC	CGCTGGCACGATGGTTCATTCT	GCCGTGGGGGGGTCTTGCTA
rs140522	CAAAGCACACACTCCATTTAATTATGTGT	AAGCACACACTCCATTTAATTATGTGC	TTCATGGACTTAAATAGATAAGTCCTCTCT

[a] Allele 1 primers have a 5’ FAM label – GAAGGTGACCAAGTTCATGCT, allele 2 primers have a 5’ VIC label – GAAGGTCGGAGTCAACGGATT

b)

SNP	PCR primer 1	PCR primer 2	Extension primer
rs34676223	ACGTTGGATGTGGTCGTAAGTGACAGAAAC	ACGTTGGATGCGAAGTTTGACTCACCCTTG	GGAGATAAAGGGGCCACTA
rs736009	ACGTTGGATGAGCTTGGTCCCAAGCTCTAC	ACGTTGGATGTGCAAACCAGTGGTTGCTA	CCCCCACCAGTGGTTGCTATCCCCA
rs41271473	ACGTTGGATGTCGTTACCTAGGATTGAGGC	ACGTTGGATGTTTAGGGAGTGGTTCCTGTG	TTTGCTTTCGGTACTGTAATAC
rs2071528	ACGTTGGATGCTGCGATTCTCGAAACCAGC	ACGTTGGATGAACCCTAATCAGAGGACCAC	GTCCGCGCTCTGCTCCC
rs71597109	ACGTTGGATGATGCTAGCGAAGGGAACATC	ACGTTGGATGTACACAAATAGGACACCAGC	CCCCACACCAGCTGGATTTC
rs57214277	ACGTTGGATGTGCTCTGAGTATGTGATGCG	ACGTTGGATGTTCTGCTTCTCCGGAGACCT	CGGAGACCTCAGGAAACTTC
rs1289214	ACGTTGGATGGAGAGCCAGCGTATATTTC	ACGTTGGATGTCTCCCTCTGAATTATTTGC	GCATATCAGTTGTCATGTGTTA
rs12654442	ACGTTGGATGAGTCTAGTGAGAAAGCTGCC	ACGTTGGATGCTCCTTCCTCCATTTTGGTG	CCCATTGTGACAGGTA
rs3800461	ACGTTGGATGGTCAGTATATCATCCACAGC	ACGTTGGATGAGTGGCCACTATGTTTTAGC	GCTGTCCTAAGTTAACCCTATAAA
rs2731567	ACGTTGGATGAGCCAGATTTGATGATGAGG	ACGTTGGATGAGGACTGCTGCAGCTGTTC	GCTGTTCTCAGCCAC
rs7026022	ACGTTGGATGGGATCATTGGATACATGCAG	ACGTTGGATGGGTTATGCCCCAGGAGTTTT	TATGACCCAGGAGTTTTTTCCATG
rs61904987	ACGTTGGATGGAAAAGCCAGCCTAGAGACG	ACGTTGGATGTAGAAGCCAACCACTAGAGC	CCTTCCTCAGCCCCTA
rs8181768	ACGTTGGATGGATAAGAGGTCTACCTCCTG	ACGTTGGATGGTAAACAAAGGCATCCTGAC	CCCTCATCCTGACTCATGCAGT
rs1036935	ACGTTGGATGAACAGACCAACCTCTTGATG	ACGTTGGATGTGCTTGCAAAGTGATGGCTG	TAATGGCCTCAAACTCTT
rs7254272	ACGTTGGATGACTCCTGGGCTTCAGTGTTC	ACGTTGGATGACCATGTAGAGCTTAGAGCC	GGAGATGGCACGATGGTTCATTC
rs140522	ACGTTGGATGTAATGCAAAGCACACACTCC	ACGTTGGATGGTGTGATTATTTTGTGGCCC	AAATAGATAAGTCCTCTCTCT

Supplementary References

1. Wellcome Trust Case Control Consortium. Genome-wide association study of 14,000 cases of seven common diseases and 3,000 shared controls. Nature 447, 661-78 (2007).

2. Berndt, S.I. et al. Genome-wide association study identifies multiple risk loci for chronic lymphocytic leukemia. Nat Genet 45, 868-76 (2013).

3. Di Bernardo, M.C. et al. A genome-wide association study identifies six susceptibility loci for chronic lymphocytic leukemia. Nat Genet 40, 1204-10 (2008).

4. Crowther-Swanepoel, D. et al. Common variants at 2q37.3, 8q24.21, 15q21.3 and 16q24.1 influence chronic lymphocytic leukemia risk. Nat Genet 42, 132-6 (2010).

5. Berndt, S.I. et al. Meta-analysis of genome-wide association studies discovers multiple loci for chronic lymphocytic leukemia. Nat Commun 7(2016).

6. Speedy, H.E. et al. A genome-wide association study identifies multiple susceptibility loci for chronic lymphocytic leukemia. Nat Genet 46, 56-60 (2014).

7. Slager, S.L. et al. Common variation at 6p21.31 (BAK1) influences the risk of chronic lymphocytic leukemia. Blood 120, 843-6 (2012).

8. Slager, S.L. et al. Genome-wide association study identifies a novel susceptibility locus at 6p21.3 among familial CLL. Blood 117, 1911-1916 (2011).

9. Sava, G.P. et al. Common variation at 12q24.13 (OAS3) influences chronic lymphocytic leukemia risk. Leukemia 29, 748-51 (2015).
